# The Role of Gangliosides in Neurodevelopment

**DOI:** 10.3390/nu7053891

**Published:** 2015-05-22

**Authors:** Kate Palmano, Angela Rowan, Rozey Guillermo, Jian Guan, Paul Mc Jarrow

**Affiliations:** 1 33 Centennial Dr, Whitianga 3510, New Zealand; E-Mail: kate.palmano@xtra.co.nz; 2 Fonterra Co-operative Group Ltd., Private Bag 11029, Palmerston North 4442, New Zealand; E-Mail: paul.mcjarrow@fonterra.com; 3 Centre for Brain Research, Auckland University, Private Bag 92019, Auckland 1142, New Zealand; E-Mails: r.guillermo@auckland.ac.nz (R.G.); j.guan@auckland.ac.nz (J.G.)

**Keywords:** gangliosides, brain, cognition, nutrition, neurodevelopment, neuroplasticity

## Abstract

Gangliosides are important components of neuronal cell membranes and it is widely accepted that they play a critical role in neuronal and brain development. They are functionally involved in neurotransmission and are thought to support the formation and stabilization of functional synapses and neural circuits required as the structural basis of memory and learning. Available evidence, as reviewed herein, suggests that dietary gangliosides may impact positively on cognitive functions, particularly in the early postnatal period when the brain is still growing. Further, new evidence suggests that the mechanism of action may be through an effect on the neuroplasticity of the brain, mediated through enhanced synaptic plasticity in the hippocampus and nigro-striatal dopaminergic pathway.

## 1. Introduction

Gangliosides are sialylated glycosphingolipids which are widely distributed throughout body tissues, principally as components of cell membranes [1]. They are expressed more predominantly in nervous tissue and are particularly abundant in brain, where they constitute 10%–12% of the lipid matter of the neuronal membrane and are largely concentrated in the grey matter [2,3,4,5,6]. Gangliosides are situated in the outer leaflet of plasma membranes [7] where they are anchored by their ceramide lipid moiety with the glycans extending into the extracellular space. They are largely localised in membrane microdomains, also known as lipid rafts, in association with other sphingolipids and cholesterol [5,8,9]. Lateral interactions within these lipid rafts and with membrane proteins play an important part in cellular processes including cytokine and adhesion signal transduction and membrane protein regulation [8,10,11]. Gangliosides contribute significantly to the cell surface glycans in neuronal cells [6,12] and constitute 75% of the conjugated sialic acid in brain [9]. They mediate functions such as cell-cell recognition through their specific sialoglycan components [10], and cell adhesion, motility and growth through glycosynaptic microdomains [13].

In humans, brain growth occurs from 12 weeks of gestation through the first three years of infancy. Adequate nutrition to support the rapid growth and development of the brain during this period is paramount [14,15]. Nutritional deprivation results in impaired development, fewer neuronal connections, impaired synaptic connectivity and irreversible consequences for cognitive function throughout life [16]. It is widely accepted that gangliosides play a critical role in neuronal function and brain development, affecting such processes as neurotransmission, neurogenesis, synaptogenesis, modulating synaptic transmission, cell proliferation, and neuronal differentiation [6,12,17,18,19,20]. Many of these processes are fundamental to the so-called neuroplasticity of the brain i.e. the ability of the brain to undergo the activity-dependent functional and morphological remodeling which subserves learning and memory [21]. While prenatal brain development mainly involves neurogenesis and migration, postnatal brain development largely involves remodeling, specifically vascular remodeling, white matter (myelin) development and synaptic development and pruning [22,23]. The most dramatic brain remodeling takes place during the first postnatal year but remodeling continues throughout life such that the mature adult brain retains considerable functional plasticity [23,24]. Further, there is mounting evidence that diet can influence brain plasticity throughout life [25].

With the growing recognition of the importance of specific nutrients in the development and maintenance of the cognitive functions of the brain, gangliosides have become a new target for infant nutrition. This review briefly summarizes the role of gangliosides in brain development, function and neuroplasticity, with emphasis on the link between dietary intake of gangliosides and effect on cognitive functioning, and special focus on the role of ganglioside supplementation in neonates. New insights into mechanisms through which dietary gangliosides may exert their effect in both young and old brain are discussed.

## 2. Structure of Gangliosides

Gangliosides consist of a ceramide core to which glycans are attached through a single glycosidic linkage at the 1-hydroxyl position [6]. The glycan chains, based on a combination of glucose, galactose and *N*-acetylgalactosamine, contain between 1–4 (and unusually up to 7) sialic residues [9] and are highly heterogeneous, resulting in high structural and functional diversity. The sialic acid ligand in brain gangliosides is exclusively of the Neu5Ac type, but this can be further modified by acetylation at the 4, 7 or 9 hydroxyl groups, with major effects on bioactivity and molecular recognition [26]. Additional heterogeneity in the ceramide moiety adds further complexity but in mammalian brain C18- and C20- sphingosine are the predominant sphingoid bases while the fatty acid ligand is typically C18 [6,9,27]. The long chain saturated fatty acid contributes to structural rigidity and increased lateral self-association of gangliosides within the outer leaflet of the membrane [9].

**Figure 1:**
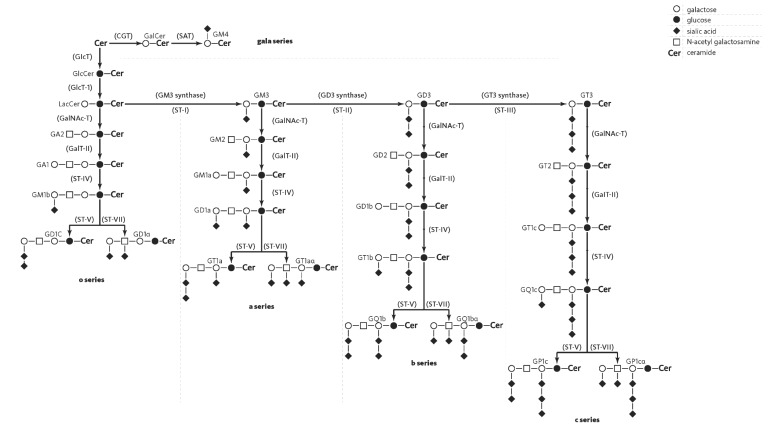
Biosynthetic pathways and nomenclature of major brain gangliosides. The gangliosides are synthesised sequentially by a series of glycosyl and sialyl (S) transferases (T).

The commonly applied Svennerholm system of ganglioside nomenclature [28] is used herein. Thus G denotes ganglioside, M/D/T/Q (mono-/di-/tri-/tetra- *etc*.) indicate the number of sialic acid residues, with an assigned number being originally based on the migration order of the gangliosides on thin layer chromatography (e.g.GM3 > GM2 > GM1; see Figure 1 for structures). Gangliosides having 0, 1, 2 or 3 sialic acid residue(s) linked to the inner galactose residue are further classified into asialo, a-, b- and c-series, respectively (see Figure 1). Subclass definition of gangliosides (and glycosphingolipids) is based on the neutral carbohydrate core attached to the ceramide (for greater detail see [6,9,20,29,30]).

## 3. Gangliosides in Human Brain

The adult brain contains ganglioside of higher complexity than other tissues [9]. During development of the brain, the pattern and expression levels of gangliosides undergo marked change, from the simple GM3 and GD3 forms predominant in the embryonic brain, to the more complex and closely related forms GM1a, GD1a, GD1b and GT1b, for which GM3 and GD3 are precursors [20,31,32]. These four complex gangliosides are the dominant species in the human brain, comprising up to 90% of gangliosides present in the adult brain [33]. Other brain gangliosides include 9-*O*-acetyl GD3 as well as the complex forms GM2, GT1a and GQ1b [18,20].

Changes in brain ganglioside expression are highly region-specific [18], appear to be tightly regulated and correlate with neurodevelopmental milestones including neural tube formation, neuritogenesis, synaptogenesis, axonogenesis and myelination [4,20,34]. A dramatic increase in brain ganglioside accretion occurs in the 10th week of gestation and continues through the first five postnatal years [35], coinciding with the most active period of myelination [36]. In particular, GM1a and GD1a are increased 12–15 fold during this period with the most active accretion being at term, coinciding with dendrite arborisation, axonal outgrowth and synaptogenesis [35]. The appearance of GM4, the third most abundant ganglioside in human white matter, occurs after the beginning of myelination [35]. Content and composition of gangliosides in the brain also change during ageing, with as much as 64% of total ganglioside being lost over 6 decades, as well as an increase in GQ1b, GT1b, and GD1b with concomitant decrease in GM1a and GD1a [37].

The change in expression levels and patterns of gangliosides during brain development is largely attributed to spatiotemporally-regulated developmental changes in the expression levels and patterns of endogenous brain glycosyltransferases. These glycosyltransferases are fundamental to ganglioside biosynthesis and in brain, as in other tissues, a series of specific glycosyltransferases catalyse the sequential addition of carbohydrate moieties to a glycosyl ceramide precursor, with complex gangliosides being synthesized sequentially from simple gangliosides in different pathways, see Figure 1, [38,39,40,41]. GM3 synthase occurs at a pivotal point in this pathway, producing the simple ganglioside GM3, which serves as a precursor to complex gangliosides of the a- and b-series that are common in the brain [40]. Conversely, ganglioside catabolism occurs stepwise via glycan-specific exoglycosidases (reviewed in [38,42,43]), with the action of sialidases providing a mechanism for functional remodeling *in vivo* [5,12].

## 4. Role of Gangliosides in Neurodevelopment and Neuro-maintenance

Early studies on isolated neurons indicated an enrichment of gangliosides in terminal axons and synaptic endings, suggesting a role in formation, development and maintenance of the nervous system [44]. The importance of gangliosides in normal functioning and integrity of the nervous system and in neurodevelopment and neural regeneration has since been demonstrated by studies with mice genetically engineered to be deficient in gangliosides expressed in both developing and mature brain (GM3, GD3, GM1a, GD1a, GD1b, GT1b). The range of structural and functional defects resulting from selective knock-out of ganglioside synthase genes included altered neural development, neuronal degeneration, loss of sensory and motor functions, demyelination, axonal deterioration, behavioral abnormalities and learning and memory loss [9,20,45,46,47]. In particular, mice expressing primarily GM3 and devoid of complex brain-type gangliosides (GM1a, GD1a, GD1b, GT1b ) suffered weight loss, progressive motor and sensory dysfunction, and deterioration in spatial learning and memory with aging [48,49]. Similarly, postnatal mice with a deficiency in GD3 and the downstream b-series gangliosides were shown to have impaired brain neurogenesis in the hippocampus resulting from a progressive loss in neural stem cell population, and exhibited depression-like behavior [46]. In humans, a defect in the GM3 synthase gene resulting in absence of GM3 and key complex gangliosides in the brain is an underlying cause of an inherited form of epilepsy which manifests not only in seizure onset in infancy, but neurological decline and blindness [50]. Many other disturbances of ganglioside metabolism in humans caused by deficiency of enzymes are associated with neurological disease, leading to uncontrolled accumulation in the brain often with severe consequences (reviewed elsewhere [9,30,51]). In addition to the well-established spectrum of disorders known as gangliosidoses that includes Tay-Sachs disease, Sandhoff disease, and GM1a gangliosidoses, GM1a and GM2 in the brain are also involved in other neurological conditions including Alzheimer’s, Huntington’s and Parkinson’s diseases [52]. There are also implications of a role of gangliosides in other conditions including infection, inflammation, insulin metabolism and cancer [52,53,54]. From a therapeutic point of view, clinical trials have shown that injected ganglioside GM1a was able to remediate symptoms of Parkinson’s disease [55] and acute ischaemic stroke [56], and also enhance neurological repair after spinal cord injury [57].

At the cellular level, the ability of exogenously applied gangliosides to potentiate neurite outgrowth in primary neurons, sensory ganglia and neuroblastoma cell lines, and axonal sprouting in regenerating axons has long been established [58,59,60,61,62,63,64]. More recently, a multifactorial mechanism for neurite outgrowth involving GM1a clustering in lipid rafts has been described [65]. Additionally, GD3 is thought to play a crucial role in maintaining the self-renewal capacity of neural stem cells, and hence neurogenesis, by sustaining EFG-induced EFGR signaling through recruitment of, and interaction with, EFGR in lipid raft micro domains [66]. The ganglioside 9-*O*-acetyl GD3 has been implicated in glial-guided neuronal migration and neurite outgrowth in both the developing and adult rat nervous system [67,68], and in facilitation of axonal growth and Schwann cell-induced myelination during both development and regeneration of mouse peripheral nerve [47]. Studies in developing human brain (gestational week 6–15) indicated GM1a involvement in glia-neuronal contacts during neuroblast migration, and GM3 localisation in cell proliferation zones [69]. Additionally, gangliosides can enhance or regulate cell survival. They have been shown to be neuroprotective against toxic insult [70], anti-apoptotic during neural cell differentiation [71], and pro-apoptotic during neural cell proliferation [72].

On another level, gangliosides have been shown to be essential for stabilising the architecture and functioning of lipid rafts, and therefore for maintenance of integrity in nerve tissue [73,74,75]. In this context, GD1a and GT1b are thought to mediate axon-myelin interactions through association with myelin-associated glycoprotein, thus helping to ensure long-term axon-myelin stabilization, axonal protection and regeneration [76,77,78].

## 5. Gangliosides in Neuroplasticity and Memory Formation

Gangliosides play a pivotal role in modulating ion channel function and receptor signaling, both of which are key functions in neuronal excitability and synaptic transmission (reviewed in [12,79]). In particular, GM1a has been implicated in maintaining neuronal viability, conduction velocity and excitability through regulation of Na^+^ channels, and in combination with GD1a, neuronal Ca^2+^ homeostasis [12,80]. In regard to synaptic transmission, exogenous GM1a and GQ1b were both shown to evoke neurotransmitter release in isolated mouse brain cortical synaptosomes through enhanced depolarisation-induced Ca^2+^ influx into the synaptosomes [81], while GQ1b was shown to be essential for synapse formation and synaptic activity in cultured rat cortical neurons [82]. A well-established model for synaptic transmission involving the Ca^2+^-binding sialic acid component of gangliosides is described elsewhere [41]. In this regard, interactions between brain gangliosides, calcium, and functional membrane proteins (ion channels, ion pumps, receptors, kinases) are thought to play a crucial modulatory role in the transmission and storage of information involved in memory [34], which forms the basis for learned behaviours and higher order cognitive processing [83]. In particular, gangliosides support the formation and stabilization of functional synapses and neural circuits required as the structural basis of memory, and in some studies, mostly employing GM1a, they have been shown to protect against age- or lesion-induced memory deficit in animals when administered exogenously (for reviews and schema see [34,41]). Synapse strength can be modified over time according to level of stimulation and it this synaptic plasticity, along with structural remodeling of neural networks activated during learning that is thought to be the underlying mechanism for learning and memory (reviewed in [21,83,84]). These cognitive functions are primarily associated with the hippocampal region of the mammalian brain and notably, there is a 30% accretion of ganglioside in the hippocampus between weeks 16 and 22 of gestation [3]. Gangliosides GM1a and GD1b have been shown to improve synaptic plasticity in the CA3 hippocampal region, a region most closely associated with spatial learning and memory [85], via prolonged post-tetanic potentiation [86]. Further, GQ1b has been functionally implicated in long term potentiation (LTP), one of the key types of synaptic plasticity in which the expression of long-lasting activity-dependent synaptic modifications occur in response to periods of high frequency stimulation [21,25,83,84,87]. In particular, GQ1b was shown to participate in activity-dependent LTP in CA1 neurons of guinea pig hippocampal slices, through modulation of NMDA receptors/Ca^2+^ channels [88], while transgenic mice with decreased levels of GQ1b exhibited learning impairment and an altered synaptic plasticity, as evidenced by an attenuation of the induction of LTP in hippocampal CA1 neurons [89]. In mice, inhibition of the biosynthesis of b-pathway gangliosides, including GQ1b, also resulted in impairment of LTP induction in hippocampal CA1 neurons, accompanied by failure to learn in a simple memory task [90]. Moreover, intraperitoneal administration of ganglioside was able to reverse the lead-induced impairment of LTP activity in rat brain [91]. *In vivo*, the relationship between both short term and long term stimulation (behavioural training) and increased expression of brain gangliosides, particularly in the hippocampus, has long been known [92,93,94]. Although exogenous administration of gangliosides into normal or functionally compromised animals has resulted in inconsistent effects on memory, behaviour and learning, possibly due to compositional differences in the ganglioside, mode of administration and different age models (reviewed in [95,96]), some of the findings nevertheless support a positive impact of gangliosides on cognitive functions. Intracerebroventricular injection of GQ1b resulted in improved spatial learning and memory in rats [97] while high doses of bovine brain ganglioside (principally GM1a, GD1a, GD1b, GT1b) administered intraperitoneally improved learning ability and memory retention in neonatal, developing and aged rats [98]. Further, exogenous GM1a improved spatial learning and memory deficits in a rat model of Alzheimer’s disease [99]. From another perspective, decrease in functional brain gangliosides induced in rats by intraventricular injection of brain ganglioside antiserum resulted in retrograde amnesia and learning deficit [100,101].

Overall, these findings support the view that the highly regulated differential expression of gangliosides appears to be closely involved in not only shaping the neural architecture of the developing brain and stabilising that architecture throughout life, but also ensuring optimum functioning and adaptation of the neural circuitry involved in neurotransmission, memory and learning.

## 6. Influences of Diet on Brain Gangliosides

The vital role of gangliosides in pre- and early postnatal development of the brain, and indeed in normal functioning and maintenance of the brain, raises the question of diet and the role it plays in providing adequate nutritional background to support appropriate expression and distribution of brain gangliosides, especially during crucial periods of brain growth, learning and assimilation. There is much evidence to support the notion that diet modulates brain structure and function, in particular brain plasticity (neurogenesis, synaptic formation and function), exerting its influence throughout the lifespan of an organism ([14,25] for review). Currently there is much interest in amelioration of age-related cognitive decline through dietary modification and in this respect, longitudinal studies have shown that frequent dairy intake is associated with better cognitive performance [102], in particular improvement in spatial working memory [103]. In neonates it has long been recognised that maternal diet throughout pregnancy and lactation can affect growth and development of the offspring, and undernutrition can have profound effects on learning and behaviour throughout life [14,15,16,104].

In rats, neonatal undernutrition induced by feeding mothers a low protein diet during lactation resulted in decreased incorporation of exogenous sialic acid into brain gangliosides and glycoprotein sialic acid, accompanied by a learning deficit which persisted into adulthood [105]. Significantly, 80% of the sialic acid injected during the brain growth period was incorporated into the synaptosomes [106]. Short term environmental stimulation in the undernourished pups resulted in improved learning coincident with improved brain ganglioside concentration, but neither parameter could be remediated to those levels found in the well-nourished control group [107]. Along with decreased ganglioside levels, postnatal undernutrition was also associated with structural deficits such as decreased brain glial cell population and reduced capacity to form myelin [108]. Lack of essential fatty acids in the maternal diet during gestation and lactation depressed both brain ganglioside and glycoprotein sialic acid concentrations and irreversibly impaired learning behaviour in the offspring [109]. Although no direct causality can be established from these studies, they nonetheless indicated that dietary-induced modifications in brain ganglioside content might impact upon cognitive functioning. Of note is another study in which similar postnatal undernutrition in rats resulted in a 55% decrease in brain gangliosides at 21 days, with a selective decrease in mono- and disialo-gangliosides [110].

The first evidence in humans that neonatal brain ganglioside concentration could be influenced by diet was provided in a study of infants who died of sudden infant death syndrome, in which it was found that those babies who had been largely breastfed had a 32% higher ganglioside content (measured as ganglioside-bound sialic acid) in their frontal cortices than formula fed babies [111]. Human milk naturally contains gangliosides, and although cow’s milk, which provides the basis for most infant formula (IF), contains similar amounts of ganglioside to human breast milk, ganglioside concentration can be substantially lower in unsupplemented IF and indeed, gangliosides may be completely absent from soy-based IF [30,112,113,114]. This implies that ganglioside intake is generally lower in IF-fed infants than in breast-fed infants. There are also some compositional differences. In human milk, GD3 is predominant in the early stages of lactation while GM3 becomes more predominant in later stages [115,116,117,118,119,120]. Minor amounts of GM1a and highly polar gangliosides are also present [121]. In bovine milk and bovine milk-based IF, GD3 is the predominant species, while GM3, GT3, GM2, GD1a and GD1b essentially comprise the remainder [116,117,122]. In both human milk and bovine milk, ganglioside levels are highest in colostrum and decline markedly through transitional to mature milk [115,116,118,123,124]. Irrespective of neonatal *de novo* contribution, this changing content and distribution of gangliosides during lactation implies an essential role of dietary ganglioside in development, with the demand being seemingly greatest during the early postnatal period.

Both observational studies [125,126,127] and clinical trial data have indicated that breast-fed infants have better cognitive performance than formula-fed babies [128,129]. This has provided the springboard for a closer examination of the micronutrient status of IF in relation to brain development and cognition, and the need to supplement IF so as to more closely match human breast milk and ensure optimum nutrition during crucial brain growth periods. Although other dietary components such as ω-3-long chain polyunsaturated fatty acids have been implicated in improved neonatal cognitive development [130,131,132], and docosahexaenoic acid (DHA) is now regularly supplemented into IF [30], the notion that dietary gangliosides may have beneficial effects in brain development and cognitive functions nevertheless remains persuasive in the light of their critical involvement in neural development and function.

An implied assumption of dietary modulation is that the dietary component(s) of interest will survive digestion, be absorbed from the gut and thence distributed to the target or effector site. It has been shown in neonatal rats that dietary ganglioside (principally GD3 in bovine milk complex lipid) can be absorbed from the intestine directly into the microdomains of enterocyte basolateral membranes, and be further distributed such as to increase total ganglioside levels in plasma, brain and retina, with increased GD3 in enterocytes and retina, but no alteration in ganglioside profile in plasma or brain [53,122,133]; and that GM1a exogenously administered to pregnant rats can distribute widely into tissues including brain, and also cross the placenta into fetal tissues [134]. Furthermore, these studies indicated that gangliosides can be remodeled post-absorption according to tissue biochemical signature and demand, and notably in brain where levels of polysialogangliosides, particularly GD1a, were increased as a result of GM1a administration [134]. This was also evidenced by studies where dietary ganglioside administered to weanling rats brought about changes in GQ1b and GM4 profiles in brain myelin and synaptomes, respectively [135]. *Ex vivo* studies have confirmed that milk-derived gangliosides GM3 and GD3 can both be transferred across the human placenta [136]. These studies provide not only evidence of the bioavailability of supplemented dietary gangliosides to both the pre-natal and postnatal brain, but also evidence that dietary gangliosides can modify tissue ganglioside composition. Further, the suggestion that the increase of GD3 in the retina resulting from dietary GD3 supplementation may stimulate neonatal retinal maturation and development [133] provides a functional link.

## 7. Dietary Gangliosides and Cognitive Functions

Both animal experiments and human clinical trials have provided some evidence that dietary gangliosides may improve cognitive functions. Rats supplemented with ganglioside-containing bovine complex milk lipid (CML; at dose rates of 0.2% and 1% of the total food intake) from an early age, through weaning and on to young adulthood showed improved cognitive measures of novelty recognition and spatial memory although brain ganglioside concentrations were not different from controls [137]. In a follow-up trial where pregnant rats were supplemented with CML at much lower doses (0.01% and 0.05% of the total food intake), GM1a, GD1a, GD1b and GT1b brain ganglioside concentrations in offspring were increased transiently around birth in the higher dose animals relative to the controls and brain weight was increased, but there were no changes in long-term behavioural functions as measured by standard behavioural tests [138]. However, the authors pointed to limitations in the trial, such as the scheduled feeding regimen which might have affected motivated learning through dopamine-induced changes in neural plasticity, and also the non-deficit model employed. In another study, ganglioside supplementation of the pre-weaning diet of artificially reared rats with CML (0.2% w/v of diet) had no effect on spatial short-term memory although the authors found the results inconclusive due to methodological considerations and in particular, lack of adequate controls. Notably, however, brain GD1b + GQ ganglioside content was significantly higher in naturally suckled pups [96]. Clearly, there are many variables to consider in design of animal trials which will adequately address the hypothesis, among them the developmental window within which gangliosides are likely to have the most impact, dose and duration of intervention and relevance in humans. In humans, the brain growth spurt begins in the last trimester of gestation and continues through to 18 months post-natally [139], whereas in rats this critical period is purely post-natal with highest rate of brain growth occurring between days 6–11 [140].

In a recent preclinical trial using neonatal piglets, in which brain growth and development is similar to humans, dietary phospholipids and gangliosides supplied as a phospholipid-rich milk protein concentrate from postnatal day 2 to day 28 improved spatial learning, moderately increased brain weight, and resulted in greater volumes in multiple brain areas along with more grey and white matter [141]. However, as brain ganglioside levels were not measured, a direct role of dietary ganglioside in the observed outcomes could not be assigned.

To date there have been few human clinical trials involving the impact of orally administered gangliosides on cognitive and other neurological functions but the outcomes have shown promise. In a large scale trial, biomedically manufactured gangliosides GM1a, GD1a, GD1b and GT1b were administered orally for 3 months or more to 2230 children suffering from cerebral palsy [142]. The authors reported that oral ganglioside treatment improved the neurological symptoms associated with cerebral palsy, in particular muscle tension, limb function, language ability and intelligence. Faster improvement was observed with younger children (0–3 years). In a small clinical study conducted to assess the impact of IF supplemented with gangliosides from CML on cognitive functions of normal healthy infants aged 0–6 months, supplementation to breast milk levels resulted in not only increased serum ganglioside levels but also improved cognitive performance on the Griffith’s scale (hand-eye co-ordination and performance IQ) when compared with an unsupplemented IF control group. Further, serum ganglioside concentration and cognitive measures in the supplemented group were very similar to a breast milk-fed reference group [143]. GM3 is the predominant ganglioside in human enterocytes and serum [144,145], and also in human breast milk after the first 3 months of lactation [42]. Although dietary gangliosides were provided principally as GD3 both in the conventional and supplemented IF, the serum ratio of GM3 to GD3 remained similar in all groups, indicating that in accordance with data from animal experiments [133], dietary ganglioside can be assimilated and remodeled following uptake into intestinal cells.

CML is a naturally rich source of gangliosides but being derived from bovine milkfat globule membrane material (see [138]) it also contains other components such as phospholipids and fatty acids which might contribute to cognitive development and memory. Cognitive improvements induced by CML supplementation cannot therefore be exclusively assigned to gangliosides. However, observed increases in serum and brain ganglioside levels following CML supplementation [133,143], coupled with lack of effect of supplementation on brain total phospholipid content [133] and provision by CML of less than effective doses of known effectors DHA and choline [138] strongly suggest the involvement of gangliosides. Moreover, [143] provide persuasive argument that the ganglioside component of CML was the likely causative agent of improved cognitive effects in their human clinical trial.

Results from other non-dietary human trials reinforce the notion that interventional gangliosides can influence neurological development and cognitive measures, as well as remediate symptoms of disease. In a small controlled trial it was found that intravenous administration of GM1a to low birth weight infants resulted in significant improvement in neurobehaviour at 6 and 12 months, in particular gross and fine movement, as assessed by the Gesell scale [146] while non-dietary GM1a was shown to diminish neurodevelopmental pathologies resulting from foetal alcohol syndrome [147,148,149]. Intracerebroventricularly administered GM1a was reported to improve cognitive function in patients with Alzheimer’s disease [150].

## 8. Mechanistic Studies of Dietary Ganglioside Effect on Cognitive Functions

**Figure 2:**
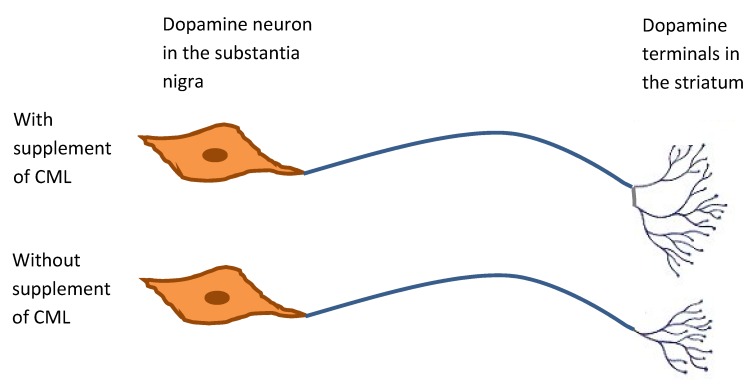
Potential mechanism for the effect of dietary ganglioside-containing complex milk lipid on learning and memory in postnatal rats. The diagram shows the nigra-striatal pathway in which the dopamine neurons project from the nigra to the striatum. Supplementation may increase the synaptic connectivity in the striatal dopamine terminals, depicted as amplification in the number of synaptic processes emerging from the terminal, and hence increase efficiency of dopamine trafficking.

In a recent study designed to probe the possible mechanisms through which ganglioside/CML supplementation might improve spatial learning and memory in postnatal rats, as observed in an earlier trial [137], our group found that CML supplementation (1% w/w of diet) may enhance neuroplasticity in the CA3 hippocampal region of young normal rats during development, possibly through enhanced synaptogenesis [151]. Our data also indicated that improved cognitive function of CML supplementation did not associate with either vascular remodeling or glutamatergic neuroplasticity in the hippocampus, striatum, and frontal cortex of young normal rats but may be associated with changes in the nigral-striatal dopaminergic pathway, which also plays a key role in learning and memory. We found an increased dopamine output in the striatum of CML-supplemented rats that was related to nigral dopamine expression, and we hypothesise that CML supplementation may result in increased synaptic transmission in the striatal dopamine terminals via an increase in the number of synaptic projections (Figure 2). The hippocampus, visual and auditory cortices and striatum undergo rapid development during early neonatal life, characterised by morphogenesis and synaptogenesis [24]. It is possible that increased synaptogenesis induced by dietary ganglioside during this rapid period of growth results in strengthened synapses and hence improved functionality. This view is supported by earlier work which showed that exogenous application of ganglioside GM1a to lesioned nigral-dopamine neurons favoured the recovery of dopaminergic synaptic function in rat striatum through increased density of striatal nerve terminal networks in unlesioned cells, via collateral sprouting [152].

In earlier work it had been found that intraperitoneal injection of GM1a improved spatial learning and memory in cognitively impaired aged rats [153], improved memory deficits in senescent rats [154] and improved spatial learning and memory deficits in a rat model of Alzheimer’s disease [99]. In our recent studies on aged rats, we found that long term dietary supplementation of CML also resulted in improved spatial learning and memory and that these benefits were associated with improved vascular density, dopamine output, and neuroplasticity in the brain regions associated with memory [155]. We speculated that CML supplementation promoted slower, but more stable, neuroplasticity in the hippocampus, leading to a more durable memory. As yet, there is little known about either the role (if any) of gangliosides in vascular remodeling or the role of vascular remodeling in learning and memory functions. The contribution of vascular remodeling to the learning and memory outcomes in the aged rats therefore remains unclear.

Glutamate is the most abundant excitatory neurotransmitter in the mammalian central nervous system and is regarded as an initiator and important regulator of synaptic plasticity, particularly through modulation of LTP (reviewed in [156]). However, dopamine, another key neurotransmitter in the brain, plays a critical role in cognitive functioning and is known to improve learning and memory formation [157,158]. The neurophysiological basis for these effects is also thought to reside in a prolonging effect on neuroplasticity, *i.e.*, through LTP [158]. Given that gangliosides have been functionally implicated in LTP, it is tempting to speculate that improvements in cognitive functions arising from the dietary ganglioside component might also be potentiated through dopamine effects on LTP neuroplasticity in aged rats.

## 9. Conclusions

Overall, the current body of research supports the idea that dietary gangliosides are important in supporting brain development early in life and further, that they might impact upon cognitive functions throughout life and crucially during infancy when there is high nutrient demand as the brain undergoes rapid remodeling. Although more research is clearly required, the elucidation of potential mechanisms through which dietary gangliosides might exert their effects on learning and memory is an interesting new development, and lends credence to the notion of nutritional benefit.

Human breast milk is a natural source of gangliosides for the neonate. Since the ganglioside content of IF is generally lower than that of breast milk, it is likely that in those cases where substitute feeding with IF is required, dietary ganglioside supply may be sub-optimal. In this case, routine supplementation of IF with gangliosides to breast milk levels to ensure optimal cognitive outcomes would seem warranted. Also, since nutrition during pregnancy is of prime importance to the developmental and cognitive outcomes of the foetus, and dietary or exogenous gangliosides are able to cross the placenta and assimilate in brain tissues, the concept of supplementing the maternal diet to ensure optimum supply of ganglioside during gestation, as has been suggested elsewhere [159], is an attractive one. Although gangliosides are found in other food products, milk products provide a convenient, natural and relatively rich source for supplementation (see [30]). In particular, bovine milk-derived CML is a food-acceptable and appropriate substrate for ganglioside fortification of IF, and safety of usage for infants has been confirmed in two recent clinical trials [143,160].
